# Supplementary material for: A public dataset of running biomechanics and the effects of running speed on lower extremity kinematics and kinetics
Source: PeerJ. 2017 May 9;5:e3298. doi: 10.7717/peerj.3298 (PMC5426356; doi:10.7717/peerj.3298)
Supplement: Supplemental Information 2 [file peerj-05-3298-s002.docx]

**Supplementary material**

This material is provided for: “**A public dataset of running biomechanics and the effects of running speed on lower extremity kinematics and kinetics”.**

**Table S1. Demographics and running characteristics of the 28 subjects.**

| Variable | Mean ± SD |
| --- | --- |
| Age (years) | 34.8 ± 6.7 |
| Height (cm) | 176.0 ± 6.8 |
| Mass (kg) | 69.6 ± 7.7 |
| Running experience (years) | 8.5 ± 7.0 |
| Average running pace (min/km) | 4.1 ± 0.4 |

*
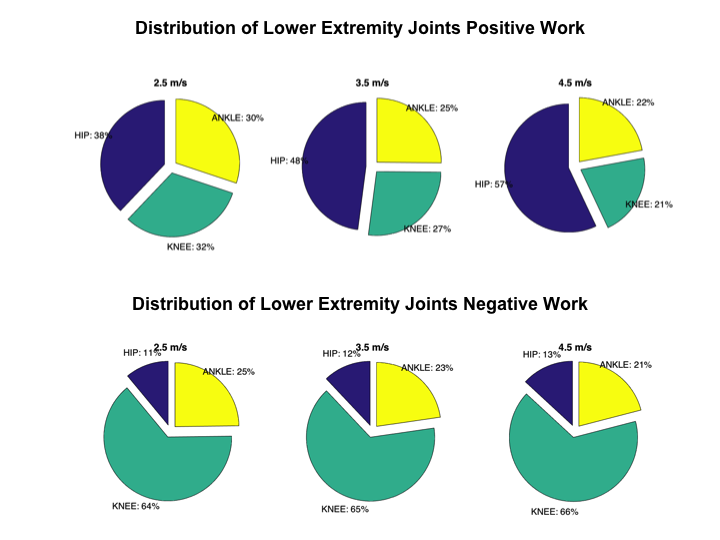
*

**Figure S1. Pie charts displaying the distribution of positive (top) and negative (bottom) joint work for the hip, knee and ankle joints during running at 2.5 m/s, 3.5 m/s and 4.5 m/s.**

**Matlab code**

**We provided an exemplary Matlab code to process the data and it is available at** [**http://demotu.org/datasets/running/**](http://demotu.org/datasets/running/)**.**
